# Effect of *Crataegus* Usage in Cardiovascular Disease Prevention: An Evidence-Based Approach

**DOI:** 10.1155/2013/149363

**Published:** 2013-12-29

**Authors:** Jie Wang, Xingjiang Xiong, Bo Feng

**Affiliations:** Department of Cardiology, Guang'anmen Hospital, China Academy of Chinese Medical Sciences, Beijing 100053, China

## Abstract

Hawthorn (*Crataegus oxyacantha*) is a widely used Chinese herb for treatment of gastrointestinal ailments and heart problems and consumed as food. In North America, the role of treatment for heart problems dates back to 1800. Currently, evidence is accumulating from various in vivo and in vitro studies that hawthorn extracts exert a wide range of cardiovascular pharmacological properties, including antioxidant activity, positive inotropic effect, anti-inflammatory effect, anticardiac remodeling effect, antiplatelet aggregation effect, vasodilating effect, endothelial protective effect, reduction of smooth muscle cell migration and proliferation, protective effect against ischemia/reperfusion injury, antiarrhythmic effect, lipid-lowering effect and decrease of arterial blood pressure effect. On the other hand, reviews of placebo-controlled trials have reported both subjective and objective improvement in patients with mild forms of heart failure (NYHA I–III), hypertension, and hyperlipidemia. This paper discussed the underlying pharmacology mechanisms in potential cardioprotective effects and elucidated the clinical applications of *Crataegus* and its various extracts.

## 1. Introduction

**Figure 1:**
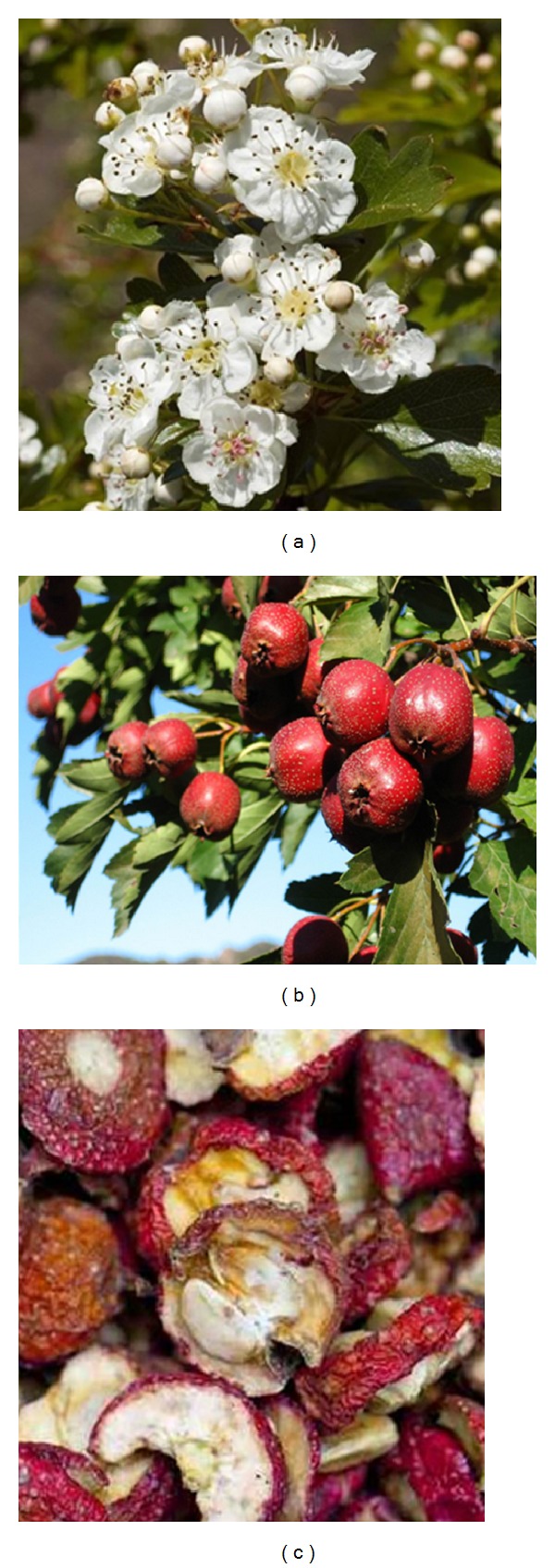
Different parts of *Crataegus monogyna* used as traditional food and folk medicine in China. (a) Flowers. (b) Ripened fruits. (c) Dried fruit for pharmaceutical use.

Hawthorn (*Crataegus oxyacantha*), also known as haw, maybush, or whitehorn, is part of a genus of spiny shrubs and trees native to temperate regions in the Northern Hemisphere in Europe, Asia, and North America [1]. It belongs to the Rosaceae family and consists of bright green leaves, white flowers, and bright red berries (as shown in Figure 1). Hawthorn has been used in folk medicine for the treatment of diarrhea, gall bladder disease, insomnia, and as an antispasmodic agent in the treatment of asthma [2]. In Chinese, hawthorn was also used for a variety of conditions including digestive problems, hyperlipidemia, poor circulation, and dyspnea [3, 4]. For example, the dried fruits are traditionally used as a digestive aid and are often made into jam, jelly, candies, or wine [5]. Also, preparations of hawthorn are available in various forms ranging from infusions and tinctures to standardized extracts and may be available variously as authorized prescription drugs, over-the-counter (OTC) medications, authorized herbal medicinal products, dietary supplements, or unregulated herbal remedies. The use of hawthorn for the treatment of cardiovascular heart disease dates back to the late 1800s [6, 7]. Current claims suggested that hawthorn could be used as an alternative therapy for various cardiovascular diseases, such as angina, hypertension, hyperlipidemia, arrhythmia, and New York Heart Association (NYHA) functional class II congestive heart failure [8, 9]. Nowadays, it is gaining attention for its potential cardiovascular enhancing and protective properties [10] and numerous laboratory tests and clinical trials have demonstrated hawthorn's efficacy in the treatment or prevention of cardiovascular diseases and the most substantial evidence for clinical benefits of hawthorn is its use in chronic congestive heart failure (CHF) [11]. A meta-analysis of randomized, placebo-controlled trials of hawthorn extract in combination with standard CHF therapy suggested several beneficial cardiovascular effects of hawthorn as compared to placebo [12]. Similarly, a 2008 Cochrane review, wherein all primary literature pertaining to the health effects of hawthorn on humans was assessed, found a significant benefit in symptom control and physiologic outcomes from hawthorn extract as an adjunctive treatment for chronic heart failure [13]. Besides, the antioxidant, positive inotropic, anti-inflammatory, and anticardiac remodeling effects and other cardiovascular protective effect of the hawthorn active ingredients were demonstrated in various in vivo and in vitro experiments. *Crataegus* has a number of pharmacological properties, but the specific mechanism is not clear.

## 2. Chemical Constituents

**Figure 2:**
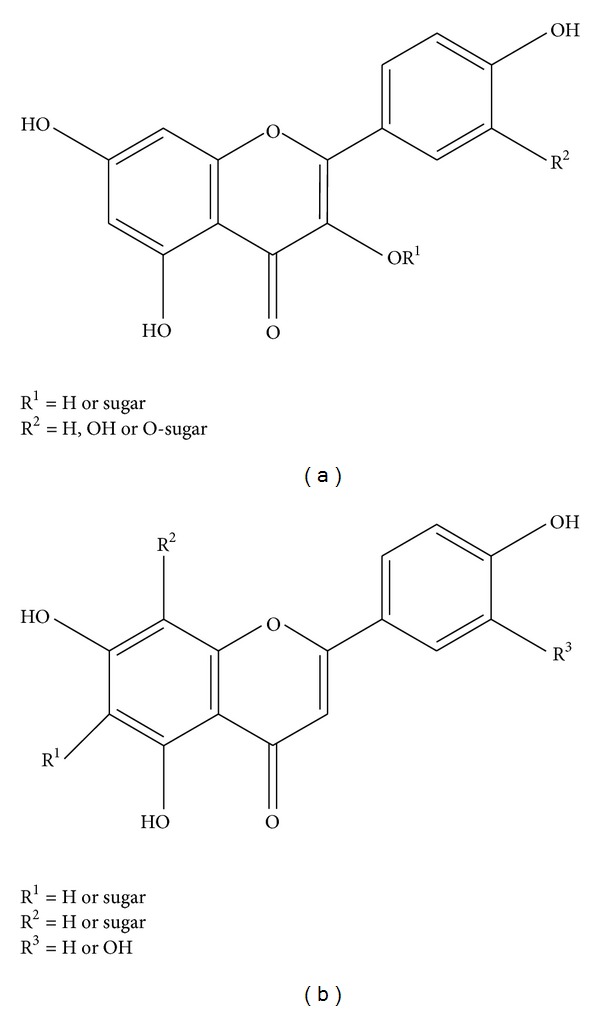
Example of flavonols (a) and flavones (b) in *Crataegus* leaves and flowers.

**Figure 3:**
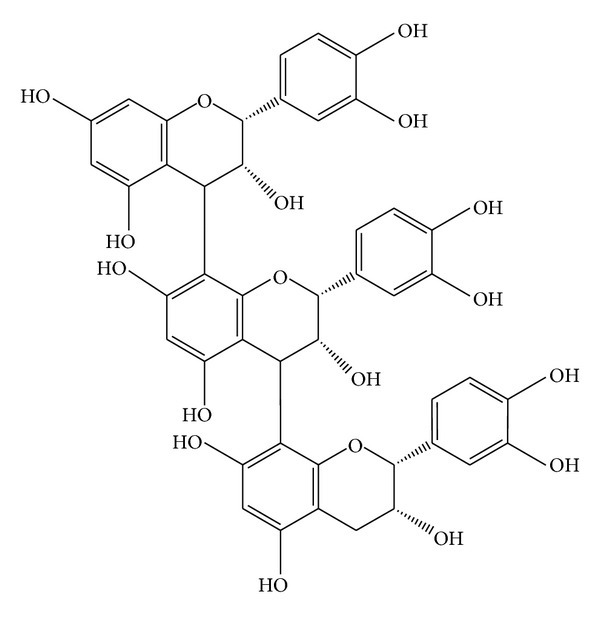
Example of an oligomeric procyanidin (OPC) consisting of three epicatechin monomers.

*Crataegus oxyacantha* is popularly known for its cardioprotective action. *Crataegus monogyna* and *Crataegus laevigata* are the major hawthorn species in middle Europe, *Crataegus pentagyna*, *Crataegus nigra*, and *Crataegus azarolus* in southern and southeastern Europe, and *Crataegus pinnatifida* and *Crataegus scabrifolia* in China [14, 15]. Available products include tinctures, tablets, teas, and aqueous extracts [16, 17]. Extracts may be prepared using hydroalcoholic (methanol or ethanol) or water-based extraction and are derived from various plant parts including, most commonly, berries or leaves and flowers [18]. The source material contains a range of pharmacologically active substances, of which the most widespread compounds reported are flavonoids, triterpenic acids, and phenol carboxylic acids [19]. Flavonoids (as shown in Figure 2) such as vitexin, hyperoside, rutin, or vitexin-2′′-O-*α*-L-rhamnoside, and catechin/epicatechin derived oligomeric procyanidins (OPC) (as shown in Figure 3) are the most important constituent. Triterpenic acids (ursolic, oleanolic, and crataegolic acids) and phenol carboxylic acids (chlorogenic and caffeic acids and various amines) are thoroughly also investigated in in vitro experiments, in animal studies, and in human clinical trials [20–23]. Currently, the most studied hawthorn extracts are WS 1442 (45% ethanol extract) and LI 132 (70% methanol extract) [24]. WS 1442 is a standardized dry extract adjusted to a content of 18.75% OPC with a starting plant material/extract ratio of 4 to 7 : 1, while LI 132 is adapted to a content of 2.2% flavonoids [25, 26].

## 3. Cardiovascular Effect 

### 3.1. Antioxidant Activity

Oxidative stress is a major concern in the pathogenesis of myocardial ischaemia. Therapeutic intervention showing antioxidant or free radical scavenging activity should exert beneficial effects against oxidative stress associated with various cardiovascular diseases (CVDs) [27]. Possible mechanisms of tincture of *Crataegus* (TCR) include preventing the increase in lipid peroxidation and activity of marker enzymes, preventing the isoproterenol-induced decrease in antioxidant enzymes in the heart, and increasing the rate of ADP-stimulated oxygen uptake and respiratory coupling ratio in isoproterenol-induced rats [28]. As we know, CVDs are associated with the structural and functional disturbances in heart mitochondria. As mitochondria produce 95% of energy necessary for heart function, therapeutic agents that could influence mitochondrial dysfunction are of special importance. Alcoholic extract of *Crataegus oxyacantha* (AEC) pretreatment maintained mitochondrial antioxidant status and prevented mitochondrial lipid peroxidative damage and decrease in Krebs cycle enzymes induced by isoproterenol in rat heart [29]. Another research showed that *Crataegus* fruit extracts decreased the mitochondrial membrane potential by 1.2–4.4 mV measured with a tetraphenylphosphonium-selective electrode and H_2_O_2_ production measured fluorometrically. Also it slightly reduced the maximal ADP-stimulated and uncoupled respiration, which might be due to inhibition of the mitochondrial respiratory chain between flavoprotein and cytochrome [30].

### 3.2. Positive Inotropic Effect

One research elucidated the potential inotropic mode of action of *Crataegus* special extract WS 1442. It is demonstrated that WS 1442 as well as its lipophilic ethyl acetate-soluble fraction A increased force of contraction in left ventricular papillary muscle strips through a cAMP-independent mechanism. As suggested by the concentration-dependent displacement of specifically bound ^3^H-ouabain from its receptor, the sarcolemmal Na^+^/K^+^-ATPase, WS 1442 seems to increase the force of contraction by inhibition of the sodium pump. Also, they can enhance the peak intracellular Ca^2+^ concentration as well in human myocardium from patients with congestive heart failure [31]. Similarly, hawthorn most probably acts on the Na^+^/K^+^-ATPase and increases the efficiency of calcium transport in cardiomyocytes [32].

### 3.3. Anti-Inflammatory Effect

Chronic and uncontrolled inflammation plays an important role in CVDs. Inflammation has been increasingly recognized as an important pathogenic component of chronic heart failure [33, 34]. Many transcriptional factors, inflammatory cytokines, enzymes, and other mediators have been shown to be related to these effects [35]. The observed anti-inflammatory effects of the water fraction of hawthorn fruit might be attributed to the downregulation of COX-2, TNF-*α*, IL-1*β*, and IL-6 expression in LPS-stimulated RAW 264.7 cells [36]. AEC most likely achieves its myocardial protection by reducing nitritive stress and oxidative stress and decreasing apoptosis. This conclusion is supported by reduced iNOS expression, nitrite levels, downregulated COX-2, decreased lipid peroxidation, decreased release of cytochrome c, and protection of DNA fragmentation [37]. Besides, hawthorn extract inhibited N-formyl-Met-Leu-Phe (FMLP-) induced superoxide anion generation, elastase release, and chemotactic migration and reduced leukotriene B4 production and lipopolysaccharide-induced generation of TNF-*α* and IL-8. Also the extract inhibited intracellular calcium signal and the extracellular calcium entry into calcium-depleted neutrophils [38]. Moreover, the anti-inflammatory mechanism also illustrated that the activity of triterpene fraction isolated from *Crataegus* was closely related to inhibition of peritoneal leukocyte infiltration and weak inhibition of phospholipase A2 (PLA2) in vitro [39].

### 3.4. Anticardiac Remodeling Effect

Cardiac remodeling comprises changes in heart structure such as alterations in cardiac wall thickness, chamber size, cell dimension, cell number, and extracellular matrix volume. These structural changes can influence heart function [40]. Hawthorn markedly reduced LV chamber volumes (VOL) after aortic constriction (AC) and augmented relative wall thickness and attenuated the AC-induced decrease in velocity of circumferential shortening (Vcfc) showing antileft ventricular remodeling and antimyocardial dysfunction in early pressure overload-induced cardiac hypertrophy [41].

### 3.5. Antiplatelet Aggregation Effect

Activated platelets play a crucial role in the pathological development of several arterial disorders, including strokes and acute coronary syndromes, which are initiated by plaque disruption and subsequent platelet-thrombus formation [42–44]. *Crataegus* extract had effective antiplatelet activity at low doses of 100, 200, and 500 mg/kg as indicated by the increase in bleeding time, decrease in platelet aggregation as assessed by PFA-100, and reduction in serum levels of thromboxane B2 [45].

### 3.6. Vasodilating Effect

Vascular protection might be associated with the direct action on endothelial cells. The endothelium regulates the contractility of the underlying vascular smooth muscle cells by releasing a number of factors, the most important of which are the nitric oxide (NO) and endothelium derived hyperpolarizing factor (EDHF). These two factors play a major role in the controlling of vascular homeostasis [46–49]. Endothelial NO-release is related to an activation of the endothelial nitric oxide synthase (eNOS) and can be stimulated by various agonists. It is concluded in vitro and vivo research that WS 1442 induced an endothelium-dependent, NO-mediated vasorelaxation via eNOS phosphorylation at serine 1177 [50]. Besides, WS 1442 induced endothelium-dependent No-mediated relaxations of coronary artery rings through the redox-sensitive Src/PI3-kinase/Akt-dependent phosphorylation of eNOS [51]. Moreover, it preserves endothelium-dependent relaxation and vascular contraction in STZ-induced diabetes, possibly by reducing iNOS expression in the aorta, by decreasing plasma levels of TNF-*α* and IL-6, and by preventing lipid peroxidation [52]. There is evidence that NO may increase activation of both the ATP-dependent K^+^-channel and the Ca^2+^-dependent K^+^-channel in vascular smooth muscle cells [53]. Similar experiment showed that procyanidins in *Crataegus* extract may be responsible for the endothelium-dependent NO-mediated relaxation, possibly via activation of tetraethylammonium sensitive K^+^ channels in isolated rat aorta [54]. Quite recently it has been demonstrated that red blood cells (RBCs) express a functional NO-synthase (rbcNOS) and rbcNOS activation has been associated with increased RBC deformability. WS 1442 activates rbcNOS and causes NO-formation in RBCs [55]. There is another opinion that hawthorn does have a vasodilating action both in the coronary circulation and the peripheral vasculature that may be mediated by inhibition of angiotensin-converting enzyme (ACE) [56].

### 3.7. Endothelial Protective Effect

Endothelial hyperpermeability, that is, a compromised endothelial barrier function, and the subsequent formation of edema are hallmarks of many severe disorders, such as atherosclerosis, asthma, sepsis, or heart failure [57–60]. One research showed that the herbal drug WS 1442 effectively protects against endothelial barrier dysfunction by its action on key determinants of endothelial permeability (adherens junctions, actin cytoskeleton, and contractile apparatus) by inhibiting the barrier-destabilizing calcium/PKC/Rho A signaling and activating the barrier-stabilizing cAMP/Epac1/Rap1 pathway [61]. Another research showed that WS 1442 prevented the deleterious hyperpermeability-associated rise of [Ca^2+^]_*i*_ by interferring with sarcoplasmic/endoplasmic reticulum Ca^2+^ ATPase (SERCA) and the inositol 1,4,5-trisphosphate (IP3) pathway without inducing store-operated calcium entry (SOCE) [62]. Past and ongoing studies also suggest that chronic intake of *Crataegus* prevented aging-related endothelial dysfunction by reducing the prostanoid-mediated contractile responses, most likely by improving the increased oxidative stress and the over expression of COX-1 and COX-2 [63].

### 3.8. Reduction of Smooth Muscle Cell Migration and Proliferation

There have been few studies on the migration and proliferation effects of herbal medications such as hawthorn. Hawthorn appears to exhibit some cardioprotective effects due to reduction of smooth muscle cell migration and proliferation properties. Currently, up to 50% of patients undergo conventional balloon angioplasty recurrent stenosis [64]. After vessel injury, biologically active components are released that trigger a dedifferentiation of vascular smooth muscle cell (VSMCs). They start to migrate and proliferate resulting in neointimal hyperplasia. Mediators involved in these processes are platelet-derived growth factor (PDGF), fibroblast growth factor (FGF), and to a lesser extent epidermal growth factor (EGF). WS 1442 decreased VSMC migration by 38% and proliferation by 44%. It inhibited VSMC DNA synthesis induced by PDGF, blocked recombinant human PDGF receptor (PDGFR)-*β* kinase activity and decreased PDGFR-*β* activation and extracellular signal-regulated kinase (ERK) activation in VSMCs [65].

### 3.9. Protective Effect against Ischemia/Reperfusion Injury

Ischemia and reperfusion (I/R) exerts multiple injuries in microcirculation, frequently accompanied by endothelial cell injury, enhanced adhesion of leukocytes, macromolecular efflux, production of oxygen free radicals, and mast cell degranulation [66]. Thus, much effort has been made to attenuate the microcirculatory disturbance by ablating one of the insults in the pathogenetic process. Preliminary research demonstrated the cardioprotective effects of hawthorn in vivo models of ischemia/reperfusion. There are at least three experiments showing the effect. Hawthorn extract WS 1442 significantly reduced the deterioration of contractile function and infarct size in rat myocardium exposed to prolonged ischemia and reperfusion [67]. Besides, it showed evident effect against reperfusion arrhythmias by reducing the average prevalence of malignant arrhythmias (VF + Flutter) and the average prevalence of ventricular tachycardia (VT) [68]. Moreover, it prevented the isoproterenol-induced decrease in antioxidant enzyme activity [69].

### 3.10. Antiarrhythmic Effect

Hawthorn extract may produce some antiarrhythmic effects in the rat heart, but the mechanism underlying the effect remains elusive. One result shows that *Crataegus* extract prolongs action potential duration and delays recovery of *V*_max⁡_ [70]. On the other hand, concerns have been raised regarding blocking repolarising potassium currents in ventricular myocytes. This effect is similar to the action of class III antiarrhythmic drugs and might be the basis of the antiarrthemic effects described for *Crataegus* extract [71]. Another mechanism showed that extract from *Crataegus* resulted in a significant decrease in the total number of ventricular ectopic beats, mainly by reduction of beats occurring as ventricular tachycardia. Also it reduced the total number of ventricular ectopic beats but this reduction was due to the decrease of single extrasystoles [72].

### 3.11. Lipid-Lowering Effect

As we know, oxidation of the low-density lipoprotein (LDL) cholesterol plays an important role in atherosclerosis [73]. This accumulation causes a cascade of inflammatory processes, resulting in an unstable atherosclerotic plaque that ultimately bursts, causing myocardial infarction [74]. Many herbs can reduce low-density lipoprotein oxidation. One research investigated the effects of seven Chinese herbs and concluded that Shan Zha (Hawthorn Fruit) is effective in lowering blood lipid levels [75]. Similar study showed that Hawthorn fruit compound lowered blood lipids in atherogenic diet fed, ApoE gene deficient atherosclerotic mice. The results showed that Hawthorn fruit compound significantly reduced the ratio between low-density lipoprotein cholesterol (LDL-C) and serum cholesterol (TC): (LDL-C)/TC, especially the triglyceride (TG) levels [76]. Besides, TCR can significantly increase the binding of ^125^I-LDL to the liver plasma membranes in vitro. This may be related to enhancement of the LDL-receptor activity. TCR was also shown to increase bile acid excretion and to depress hepatic cholesterol synthesis in atherogenic diet fed rats by upregulating hepatic LDL-receptors resulting in greater influx of plasma cholesterol into the liver [77]. Treatment using hawthorn fruit can decrease serum cholesterol that involves the inhibition of cholesterol absorption mediated by downregulation of intestinal acyl CoA: cholesterol acyltransferase (ACAT) activity in Caco-2 cells. In animals research, hawthorn significantly lowered plasma non-HDL (VLDL + LDL) cholesterol concentrations and decreased hepatic cholesterol ester content [78]. The flavonoids fraction showed inhibitory effects on TG and glucose absorption and accelerating effects on gastrointestinal transit in vivo and suppressed the accumulation of TG and free fatty acid. It also suppressed the gene expressions of C/EBP*α*, PPAR*γ*, SREBP 1c, aP2, and adiponectin in vitro [79]. As we know, LPL plays an important role in lipoprotein metabolism and is expressed in various tissues, especially adipose and muscle tissue, where it plays different roles. Hawthorn flavonoids increase LPL expression through a PPAR*γ*-dependent mechanism directed towards identification of the components [80]. TCR prevented the elevation of lipids in the serum and heart and caused a significant decrease in lipid accumulation in the liver and aorta reverting the hyperlipidemic condition of these rats. The extract significantly restored the activity of antioxidant enzymes such as superoxide dismutase, catalase, glutathione peroxidase, and glutathione, thereby restoring the antioxidant status of the organism to almost normal levels [81]. One research used the larval Zebrafish as model to test plant-based dietary intervention of hypercholesterolemia and it was demonstrated that hawthorn leaves and flowers have the potential to affect cardiac output as well as intravascular cholesterol levels [82].

### 3.12. Decrease of Arterial Blood Pressure Effect

It was observed that *Crataegus*, especially the hyperoside fraction, prevented L-NAME-induced hypertension in rats and had beneficial effects on the cardiovascular system [83]. *Crataegus* administered at escalating doses produced a dose-time-dependent decrease in heart rate (HR) and mean arterial pressure (MAP). Higher doses produced the most significant reduction in both HR and MAP and induced sinus node suppression and progressive atrio-ventricular blockade. The underlying mechanism appeared to be related to the direct stimulation of the muscarinic receptor M2 and possible blockade of beta-receptors, while the hypotension was caused by enhanced nitric oxide release [84]. (All above cardiovascular effects were shown in Tables 1 and 2).

## 4. *Crataegus* for Clinical Cardiovascular Disease Prevention 

CVD are considered a serious health public problem due to the high morbidity and mortality rates. It caused 17.1 million deaths yearly worldwide according to the World Health Organization (WHO) [85]. Role of *Crataegus* in CVD prevention has been a topic of concerns for many years. However, these claimed benefits were not supported by evidence-based clinical studies. In recent years, *Crataegus* has been a focus of attention because of its potential role in the prevention of various aspects of cardiovascular disease. Evidence from numerous studies suggests that *Crataegus* works through various mechanisms to achieve this favorable effect. Majority of the studies have shown positive impact for various CVD; however, one contradictory study showed that CSE does not reduce heart failure progression, even to increase the early risk of HF progression [86]. This paper critically examines the current scientific literature concerning claims of cardiovascular benefits from *Crataegus* and its extract since 1990. We searched all human studies of clinical trials in English assessing the effect of *Crataegus* on cardiovascular disease prevention among patients (congestive heart failure, hyperlipidemia, hypertension, or c Arrhythmias were included) in five major electronic databases, including CNKI, CBMdisc, VIP, PubMed, and the Cochrane Library, to retrieve any potential randomized controlled trials (RCTs). A number of keywords were used for data searching including *Crataegus* and cardiovascular disease clinical trial, *Crataegus* hypertension and hyperlipidemia, *Crataegus* arrhythmias platelet aggregation, and clinical trial. Finally, there were 15 trials included in the review, of which 8 trials were therapies of heart failure, 4 trials were therapies of hypotension, and 3 trials of hyperlipidemia (as shown in Table 3).

### 4.1. CHF

Contemporary therapies of heart failure such as ACE inhibitors, beta-blockers, spironolactone, implantable cardioverter defibrillators, and biventricular pacemakers have produced remarkable reductions in morbidity and mortality. However, quality of life (QOL) for patients with heart failure remains impaired and improved treatment regimens are still needed. *Crataegus* extract is an adjunct to conventional treatment in patients with HF (New York Heart Association classes I–III) due to its positive inotropic, antiarrhythmic, and vasodilating properties. The hawthorn extract may provide additional benefit in symptoms control (fatigue, listlessness, dyspnoea under strain, pretibial oedema, and rapid exhaustion) and frequency of nocturnal urinations and exercise tolerance (distance walked and number of stairs ascended without fatigue) [88]. A number of randomized, controlled trials were carried out to study the effect of different preparation of *Crataegus* on congestive heart failure. The majority of hawthorn clinical trials have been performed with WS 1442, a dry extract from hawthorn leaves with flowers (4–6.6 : 1), extraction solvent ethanol 45% (w/w).

In a 3-year open cohort study, 372 patients (261 females and 111 males) of stage NYHA II taking 900 mg/day WS 1442 in addition to their standard medication were followed for three years by their treating office-based physicians. Outcome parameters demonstrated that maximal workload (MWL), left ventricular ejection fraction (LVEF) and pressure-heart rate product increase (PHRPI) at 50 W ergometric exercise improved more in active treatment than in placebo patients. In addition improvement of typical symptoms like reduced exercise tolerance, exertional dyspnea, weakness, fatigue, and palpitations improved more with active treatment and in patients with more severe symptoms [89]. Similarly, a multicentre nonrandomized cohort study in patients aged 50–75 years in received Cralonin (*n* = 110) or ACE inhibitorydiuretics (*n* = 102) for 8 weeks. The trial using *Crataegus* preparation Cralonin among NYHA class II. Patients demonstrated that the Cralonin is noninferior to usual ACE inhibitorydiuretics treatment for mild cardiac insufficiency on all parameters except BP reduction [90].

Another placebo-controlled, randomized, parallel group, multicentre trial recruiting 143 patients and treated with 3 times 30 drops of the extract (*n* = 69) or placebo (*n* = 74) for 8 weeks showed the efficacy and safety of a standardised extract of fresh berries of *Crataegus oxyacantha* L. and *monogyna* Jacq. (*Crataegisan*) in patients with cardiac failure NYHA class II. The result is confirmed that changes in blood pressure-heart rate product (BHP) at 50 watts and at comparable maximum load were in favour of *Crataegus* extract but the results are not statistically significant. What is more, an improvement in their heart failure condition may be achieved under long term therapy [91]. Effective therapy in patients was also seen in other clinical trials. In a randomized, placebo-controlled, double-blind clinical study for 12 weeks with either WS 1442 (*n* = 20) or placebo (*n* = 20), the difference between the groups was borderline statistically significant in the exercise tolerance and the double product (heart rate × systolic blood pressure × 10^−2^). It is demonstrated that WS 1442 was safe and well tolerated and was clinically effective in patients with congestive heart failure corresponding to NYHA class II [92].

One RCT studied effect of the extract LI 132 on chronic heart failure defined as NYHA functional class II. Patients were treated either with *Crataegus* extract (*n* = 50) or with a placebo preparation (*n* = 50) for a period of 8 weeks, with a wash-out phase of one week. Outcomes of MWL, LVEF, PHRPI and typical symptoms were statistically significant with the LI 132 preparation compared to the patients treated with the placebo preparation. Apart from that, a significant reduction of the systolic blood pressure, of the heart rate and of the pressure/rate product was observed for the patients treated with the verum preparation. Besides, there were no severe side effects observed [93].

However, controversy result showed that hawthorn provides no symptomatic or functional benefit when given with standard medical therapy to patients with heart failure. The research performed a randomized, double-blind, placebo-controlled trial in 120 ambulatory patients with NYHA class II-III chronic heart failure. All patients were randomized to either hawthorn 450 mg twice daily or placebo for 6 months. But there were no significant differences between groups in the change in 6 min walk distance or on measures of QOL, functional capacity, neurohormones, oxidative stress, or inflammation [94].

Two clinical trials used WS 1442 to investigate the efficacy and safety of an add-on treatment in patients with congestive heart failure. In one of this randomized, double-blind, placebo-controlled multicenter study, 2681 patients (WS 1442: 1338; placebo: 1343) were included. Results showed that WS 1442 reduced sudden cardiac death by 39.7%, so that it was safe to be used in patients receiving optimal medication for heart failure [95]. Another research included 209 patients randomized to treatment with 1800 mg of WS 1442, 900 mg of WS 1442, or with placebo for 16 weeks. The data from the study confirm that there is a dose-dependent effect of WS 1442 on the exercise capacity of patients with heart failure and on typical heart failure-related clinical signs and symptoms. The maximal tolerated workload during bicycle exercise showed that increase and typical heart failure symptoms as rated by the patients were reduced to a greater extent by WS 1442 than by placebo, so that the drug was shown to be well tolerated and safe [87]. To ascertain the effectiveness of *Crataegus* in CHF therapy, meta-analyses are required to prove its efficacy.

### 4.2. Hypertension

Hypertension is an increasingly important medical and public health issue, which could lead to severe complications [96]. It is an important risk factor for CVDs. Currently, it affects 1 billion people worldwide, and this number is expected to rise to 1.6 billion by 2025 [97, 98]. Although there have also been significant advances in therapeutic concepts and measures; however, hypertension in most individuals remains untreated or uncontrolled. With the popularity and prevalence of Chinese medicine (CM), there has been a growing interest in Chinese herbal medicine (CHM) for patients with hypertension both in China and the West [99–104]. Several small clinical trials with hawthorn have demonstrated modest blood pressure reduction. Randomised controlled trial was designed to investigate the effects of hawthorn for hypertension in type 2 diabetes patients who were randomized to daily 1200 mg hawthorn extract (*n* = 39) or placebo (*n* = 40) for 16 weeks, taking prescribed drugs. Results demonstrated that there was a significant group difference in mean diastolic blood pressure reductions: the hawthorn group showed greater reductions than the placebo group. What is more, this is the first randomized controlled trial to demonstrate a hypotensive effect of hawthorn in patients with diabetes taking medication [105].

One pilot study was aimed at investigating the hypotensive potential of hawthorn extract and magnesium dietary supplements individually and in combination, compared with a placebo. Volunteers were then randomly assigned to four groups: 600 mg hawthorn extract, 500 mg hawthorn extract, a combination of the previous two groups and placebo. Results showed that there was a decline in both systolic and diastolic blood pressure in all treatment groups or placebo, but hawthorn extract group showed a promising reduction in the resting diastolic blood pressure at week 10 in the 19 subjects, compared with the other groups. Furthermore, a trend towards a reduction in anxiety was also observed in those taking hawthorn compared with the other groups [106].

Similarly, in order to test the efficacy of a camphor-*Crataegus* berry combination (CCC) on orthostatic hypotension, two similar, controlled, randomized studies were carried out in a balanced crossover design in 24 patients each with orthostatic dysregulation. Results showed that CCC drops decreased the orthostatic fall in blood pressure, especially affecting diastolic blood pressure after 1 minute of orthostasis in all dosages as compared to placebo. A statistically significant effect of the highest dose of 80 drops on diastolic blood pressure could be demonstrated after 1-, 3-, and 5-minute orthostasis [107].

Clinical investigations exploring the effects of *Crataegus* and its various preparations in hypertension have demonstrated somewhat contradictory results. One randomized, controlled cross-over designed trial was to investigate the relationship between hawthorn extract dose and brachial artery flow mediated dilation (FMD), an indirect measure of nitric oxide release. Randomly sequenced doses of hawthorn extract (1000 mg, 1500 mg, and 2500 mg) and placebo were assigned to each participant. However, results showed that there was no evidence of a dose-response effect for our main outcome (FMD percent) or any of our secondary outcomes, such as absolute change in brachial artery diameter and blood pressure [108].

### 4.3. Hyperlipidemia

Although the lipid-lowering property of the hawthorn extract has been shown in a number of animal studies by means of reducing in total cholesterol, low density lipoprotein, and ApoB synthesis, there are still few well-designed clinical trials. One study included 49 diabetic subjects with chronic CHD who were randomly assigned to either a micronized flower and leaf preparation of *C. laevigata* group or a matching placebo. The main results were that *C. laevigata* decreased NE and showed a trend to lower LDL-C compared to placebo as add-on treatment for diabetic subjects with chronic CHD [109]. Two Chinese clinical trials used Shan Zha Jingjiangzhi pill as therapy drugs compared with Duoxokang pill and placebo, separately. Results showed that, compared with Duoxikang pill, Shanzha Jingjiangzhi pill can lower TG and TC [110]. While compared with placebo, more benefits about decreasing TC, TG, and LP(a) and increasing HDL-C were attained from Shanzha Jingjiangzhi pill [111].

## 5. Dosage and Side Effects

Then what is the adverse effect of *Crataegus*? How to use the hawthorn properly? Although modern drugs are effective in preventing cardiovascular disorders, their use is often limited because of their side effects [112]. Most of the adverse events were mild to moderate and majority of studies indicate that oral hawthorn is well tolerated. One systematic review included 29 clinical studies of 7311 patients. Overall, 166 adverse events were reported. Eight severe adverse events have been reported with the LI 132 extract such as dizziness/vertigo, gastrointestinal complaints, headache, migraine, and palpitation. Hawthorn is a slow-acting herb and should be used for at least 4 to 8 weeks for full benefit. The dosage depends on the type of preparation and source material [113]. Most effective dosage was unknown currently. Recommended dosages range from 160 to 1800 mg per day in two or three divided days. There were no reports of drug interactions.

## 6. Conclusions and Perspectives

In the last 20 years, over 60% of new drugs for the treatment of cancer and 75% of new drugs used to treat infectious diseases were of natural health products [114]. In North America, however, natural health products (NHPs) are considered as food and dietary supplements and are therefore sold in health food stores [115]. In North America, and Canada in particular, NHPs are considered mainly used in the treatment of heart-related problems [116]. *Crataegus* and its various extracts were such NHPs. Currently, *Crataegus* products are currently marketed as an alternative treatment for hypertension, angina, arrhythmia, and the early stages of congestive heart failure by regulating whole body on multilevel and multitargets. What is the mechanism of *Crataegus* for cardiovascular diseases? The previous described animal studies have suggested that hawthorn extracts exert a wide range of cardiovascular pharmacological properties, including antioxidant activity, positive inotropic effect, anti-inflammatory effect, anticardiac remodeling effect, antiplatelet aggregation effect, vasodilating effect, endothelial protective effect, reduction of smooth muscle cell migration and proliferation, protective effect against ischemia/reperfusion injury, antiarrhythmic effect, lipid-lowering effect, and decrease of arterial blood pressure effect. Moreover, numerous clinical studies have demonstrated that hawthorn preparations are very effective in early stages of congestive heart failure. A few researches were reported on therapy of hypertension and hyperlipidemia.

In China, *Crataegus* was first mentioned in “New Materia Medica.” The herb was used widely in traditional Chinese medicine, particularly in department of internal medicine, such as the food stagnation, nausea or vomiting, abdominal pain or diarrhea, hernia pain, hematocele in bosom, and postpartum lochia. Seldom narration was seen in ancient literature on *Crataegus* for treatment of cardiovascular diseases but many literature showed that *Crataegus* had the effect of “activating blood and dissolving stasis.” Since *Crataegus* has effect of eliminating food mass and removing blood stasis, it can be used for treatment of stomach disease and cardiovascular disease. In traditional Chinese medicine, there is a theory of “Treating heartache by regulating the spleen and stomach.” It comes from “gastric collaterals goes into heart” in the ancient literature of “The Miraculous Pivot.” The theory regarded that chest-bi had close association with dysfunction of spleen and stomach in physiological and pathological aspects, so it is important for treating heartache by regulating the spleen and stomach. In future, carrying out the research of traditional Chinese medical theory combined with modern pharmacological achievement is beneficial to the treatment of heart disease.

Nowadays, with the population of NHPs, finding the high efficiency and fewer adverse effects of cardiovascular-protective drugs from Chinese herb and formulas attracts great attention of researchers, and the study of target or mechanism of Chinese herb and formulas for hypertension is to be the hot topic of research and development of antihypertensive drugs. But there are still some problems we need to arise. On current, anminal research of *Crataegus* on vasodilating effect and lipid-lowering effect were performed more frequently than those other effect of studies. Nevertheless there are only a few studies that have been published about the anticardiac remodeling effect and effect of reducting smooth muscle cell migration and proliferation. So, further systematic in vivo and in vitro researches are warranted to explore and verify the potential effect to provide precise guidance for clinical use and new drug discovery. Besides, with the studies published, the strength of the evidence, however, was often limited by lack of controls or placebos, nonrandomization, non-blinded design, or small numbers of patients. It is imperative to conduct multicentered, large-sized samples and randomized and arid controlled trials to reasonably evaluate the efficacy and safety of Chinese herb and formulas for CVDs. In addition, there are so many active ingredients in *Crataegus*, so that large quantity of active ingredients should be identified, extracted, and purified from the herb. What is more, some active ingredients are chemically unstable, which have limited the large-scale synthesis. All these pressing issues should be resolved in future researches.

## Figures and Tables

**Table 1:** Compounds derived from *Crataegus* in vivo cardiovascular effects.

Target	Compounds	Animal/organs	Effect	References
Antioxidant effect	TCR	Rat heart	Lipid peroxidation;	Jayalakshmi and Devaraj, 2004 [28]
			Activity of marker enzymes;	
			Antioxidant enzymes;	
			Oxygen uptake;	
			Respiratory coupling ratio	

Positive inotropic effect	WS 1442	Human myocardial tissue	cAMP-independent mechanism;	Schwinger et al., 2000 [31]
			Sarcolemmal Na^+^/K^+^-ATPase;	
			Sodium pump;	
			Intracellular Ca^2+^ concentration;	

Anti-inflammatory effect	AEC	Rat heart	Nitritive stress;	Vijayan et al., 2012 [37]
			Oxidative stress;	
			iNOS expression;	
			COX-2;	
			Lipid peroxidation;	
			Cytochrome c;	

Anti-cardiac remodeling effect	WS 1442	Rat	LV chamber volumes (VOL);	Hwang et al., 2008 [41]
			Relative wall thickness;	
			Vcfc;	

Antiplatelet aggregation	*Crataegus aronia* syn. Azarolus (L)	Rat blood	Bleeding time;	Abdullah et al., 2012 [45]
			Platelet aggregation;	
			Serum levels of TXB2;	

Vasodilating effect	WS 1442	Isolated rings of rat aorta	Endothelium-dependent,	Brixuis et al., 2006 [50]
			NO-mediated vasorelaxation;	
			eNOS phosphorylation;	
	*Crataegus microphylla* CM extract	Rat thoracic aorta	Endothelium-dependent relaxation;	Gökçe et al., 2013 [52]
			Vascular contraction;	
			iNOS expression;	
			Plasma levels of TNF-*α*, IL-6;	
			Lipid peroxidation;	
	CE	Rat aorta	Endothelium-dependent nitric oxide(NO)-mediated relaxation;	Kim et al., 2000 [54]
			Tetraethylammonium-sensitive K^+^ channels;	

Endothelial protection	WS 1442	Rat main mesenteric arteries	Prostanoid-mediated contractile responses;	Idris-Khodja et al., 2012 [63]
			Oxidative stress;	
			COX-1 and COX-2;	

Protect I/R injury	WS 1442	Rat myocardium	Contractile function;	Veveris et al., 2004 [67]
			Infarct size;	
	*Crataegusoxyacantha*	Rat heart	Average prevalence of malignant arrhythmias (VF + Flutter);	Makdessi et al., 1999 [68]
			Average prevalence of VT;	
	TCR	Rat heart	Antioxidant enzyme activity;	Jayalakshim and Devaraj, 2004 [69]

Antiarrhythmic effect	*Crataegus meyeri* extracts	Rat	Ventricular ectopic beats;	Garjani et al., 2000 [72]
			Single extrasystoles;	

Lipid-lowering effect	HFC	Mice	Blood lipids;	Xu et al., 2009 [76]
			(LDL-C)/TC;	
			TG levels;	
	HFC	Hamsters	Serum cholesterol;	Zhang et al., 2002 [78]
			VLDL + LDL cholesterol;	
			Hepatic cholesterol ester content;	
	The leaf of *Crataegus pinnatifida *	Mice	TG and glucose absorption;	Wang et al., 2011 [79]
			Gastrointestinal transit;	
			TG and free fatty acid;	
	Hawthorn flavonoids	Mice	LPL expression;	Fan et al., 2006 [80]
			PPAR*γ*-dependent mechanism;	
	TCR	Rat	Lipids in the serum and heart;	Akila and Devaraj, 2008 [81]
			Lipid in liver and aorta;	
			Activity of antioxidant enzymes;	
	Whole plant of *Crataegus *	Zebrafish	Intravascular cholesterol levels;	Robert et al., 2012 [82]

Decrease of arterial blood pressure	*Crataegus tanacetifolia* leaf extract	Rats coronary artery	L-NAME-induced hypertension;	Koçyõldõz et al., 2006 [83]
			Beneficial effects on cardiovascular system;	
	Aqueous extract of *Crataegus *	Rat	HR and MAP;	Shatoor, 2013 [84]
			Sinus node suppression;	
			Atrioventricular blockade; Muscarinic receptor M2;	
			Blockade of beta-receptors;	

Notes: TCR: tincture of *Crataegus*; AEC: alcoholic extract of *Crataegus oxycantha*; Vcfc: velocity of circumferential shortening; TXB2: thromboxane B2; CE: *Crataegus* fruit extracts; CM: *Crataegus microphylla*; VT: ventricular tachycardia; TC: serum cholesterol; LDL-C: low-density lipoprotein cholesterol; TG: triglyceride; HFC: hawthorn fruit compound; HR: heart rate; MAP: mean arterial pressure.

**Table 2:** Compounds derived from *Crataegus* in vitro cardiovascular effects.

Target	Compounds	Cell/tissues	Effect	References
Antioxidant effect	AEC	Mitochondria from rat heart	Mitochondrial lipid peroxidative damage;	Jayalakshim et al., 2006 [29]
			Kreb's cycle enzymes;	
	CE	Mitochondria from rat heart	Mitochondrial membrane potential;	Bernatoniene et al., 2009 [30]
			H_2_O_2_ production;	
			Maximal respiration;	
			Mitochondrial respiratory chain;	

Positive inotropic effect	Two alcohol extracts	Neonatal rat cardiomyocytes	Na^+^/K^+^-ATPase;	Rodriguez et al., 2008 [32]
			Calcium transport;	

Anti-inflammatory effect	Water fraction from hawthorn fruit	LPS-stimulated RAW 264.7 cells	COX-2, TNF-*α*, IL-1*β*, and IL-6 expression;	Li and Wang, 2011 [36]
	Hawthorn extract	Human blood neutrophils	Superoxide anion generation;	Dalli et al., 2008 [38]
			Elastase release;	
			Chemotactic migration;	
			Leukotriene B4 production;	
			TNF-*α* and IL-8;	
			Intracellular calcium signal;	
	Triterpene fraction isolated from *Crataegus *	Peritoneal exudates	Peritoneal leucocyte infiltration Phospholipase A2;	Ahumada et al., 1997 [39]

Vasodilating effect	WS 1442	HCAEC	eNOS phosphorylation;	Brixuis et al., 2006 [50]
	WS 1442	Porcine coronary artery endothelial cells	Src/PI3-kinase/Akt-dependent phosphorylation of eNOS;	Anselm et al., 2009 [51]
	Hawthorn extract	VSMCs	ATP-dependent K+-channel;	Waldron and Cole, 1999 [53]
			Ca^2+^-dependent K^+^-channel;	
	WS 144	Human venous blood cell	rbcNOS and NO-formation;	Rieckeheer et al., 2011 [55]

Endothelial protection	WS 1442	HUVECs	Endothelial permeability;	Bubik et al., 2012 [61]
			Calcium/PKC/Rho A signaling pathway;	
			cAMP/Epac1/Rap1 pathway;	
	WS 1442	HUVECs	Hyperpermeability-associated rise of [Ca^2+^]_i_;	Elisabeth et al., 2012 [62]
			SERCA and IP pathway;	

Reduction of smooth muscle cell migration and proliferation	WS 1442	Rat aortic VSMCs	VSMC migration and proliferation;	Fürst et al., 2010 [65]
			VSMC DNA synthesis;	
			PDGFR-*β* kinase activity;	
			ERK activation;	

Antiarrhythmic effect	LI 132	Guinea pig ventricular myocytes	Block repolarizing potassium currents;	Müller et al., 1999 [71]

Lipid-lowering effect	TCR	Rat liver plasma membranes	Binding of ^125^I-LDL to the liver plasma;	Rajendran et al., 1996 [77]
			LDL-receptor activity;	
			Increase bile acid excretion;	
			Depress hepatic cholesterol synthesis;	

Lipid-lowering effect	HFC	Caco-2 cells	ACAT activity;	Zhang et al., 2011 [78]
	The leaf of *Crataegus pinnatifida *	3T3-L1 cells	Gene expressions of C/EBP*α*, PPAR*γ*, SREBP 1c, aP2 and adiponectin	Wang et al., 2011 [79];

Notes: AEC: alcoholic extract of *Crataegus oxycantha*; CE: *Crataegus* fruit extracts; ACAT: acyl CoA (Coenzyme A): cholesterol acyltransferase; HCAEC: human coronary artery endothelial cells; HUVECs: human umbilical vein endothelial cells; SERCA: sarcoplasmic/endoplasmic reticulum Ca^2+^ ATPase; IP3: inositol 1,4,5-trisphosphate; ERK: extracellular signal-regulated kinase; HFC: hawthorn fruit compound; NYHA: New York Heart Association.

**Table 3:** Randomized, controlled, double-blind trials of Hawthorn extract for cardiovascular diseases.

Study	Design^a^	Target	Duration	Dose	Case/control	Primary outcome measures
Eggeling et al., 2011 [89]^b^	OPC	Early chronic heart failure	156 w	900 mg, qd	372/—	Improve outcomes of MWL, LVEF, PHRPI, BP, HR, DP, and typical symptoms.

Schröder et al., 2003 [90]^e^	Double-blind, Nonrandomized controlled trial	Mild cardiac insufficiency (NYHA II)	8 w	100 mL, tid	110/102	Change HR, BP, DP, symptoms, frequency of nocturnal urinations, and exercise tolerance.

Degenring et al., 2003 [91]^c^	RCT, pg	Congestive heart failure (NYHA II)	8 w	2.25 mL, qd	69/74	Change BHP and maximum load.

Zapfe Jun, 2001 [92]^b^	RCT, pg	Congestive heart failure (NYHA II)	12 w	240 mg, qd	20/20	Increase exercise tolerance and reduce the DP.

Schmidt et al., 1994 [93]^d^	RCT, pg	Congestive heart failure (NYHA II)	8 w	600 mg, qd	50/50	Reduce the SBP, HR, and DP.

Zick et al., 2009 [94]^b^	Randomized controlled trial	Chronic heart failure (NYHA II-III)	24 w	450 mg, bid	60/60	No symptomatic or functional benefit when given with standard medical therapy.

Holubarsch et al., 2008 [95]^b^	RCT, pg	Chronic heart failure (NYHA II/III)	48 w	900 mg, qd	1338/1343	Reduce the incidence of sudden cardiac death.

Tauchert, 2002 [87]^b^	RCT, pg	Congestive heart failure (NYHA III)	16 w	900/1800 mg, qd	70/69	The treatment is safe and well tolerated.

Belz et al., 2002 [105]^e^	RCT, pg	Hypertension	16 w	1200 mg, qd	39/40	Lower mean DBP.

Walker et al., 2006 [106]^e^	RCT, pg	Hypertension	10 w	500 mg, qd	19/17	Lower both SBP and DBP, especially DBP.

Walker et al., 2002 [107]^e^	RCT, co	Hypertension	5 min.	80 drops, qd	24/24	Lower DBP.

Asher et al., 2012 [108]^e^	RCT, co	Hypertension	3 d	1000/1500/2000 mg, bid	15/6	No evidence of a dose-response effect of hawthorn extract on FMD.

Dalli et al., 2011 [109]^e^	RCT, pg	Hyperlipidemia	24 w	400 mg, tid	24/21	Decrease NE and lower LDL-C.

Liang and ye, 2004 [110]^e^	Randomized controlled trial	Hyperlipidemia	5 w	60 mg, tid	60/52	Decrease TC, TG, LDL-C.

Shen et al., 2000 [111]^e^	Clinical controlled trial	Hyperlipidemia	4 w	5 pills, tid	120/20	Decrease TC, TG, and LP(a) and increase HDC.

^a^RCT: randomized, double blind, placebo-controlled trial; co: crossover; pg: parallel group; OPC: open prospective cohort study; ^b^ *Crataegus* extract WS 1442; ^c^Crataegisan; ^d^ *Crataegus* extract LI 132; ^e^Other extracts or preparations of *Crataegus*; MWL: maximal workload; LVEF: left ventricular ejection fraction; PHRPI: pressure-heart rate product increase; BHP: blood pressure-heart rate product; HR: heart rate; BP: blood pressure; DP: double product (evaluated on a bicycle ergometric test and defined as heart rate × systolic blood pressure × 10^−2^ where HR is heart rate in bpm and BP blood pressure in mmHg); NYHA: New York Heart Association; SBP: systolic blood pressure; DBP: diastolic blood pressure; NE: neutrophil elastase; LDL-C: LDL cholesterol; FMD: flow mediated dilation.

## Abbreviations

AC: Aortic constriction
ACAT, acyl CoA: Cholesterol acyltransferase
ACE: Angiotensin-converting enzyme
AEC: Alcoholic extract of *Crataegus oxyacantha *
BHP: Blood pressure-heart rate product
BP: Blood pressure
CVD: Cardiovascular diseases
CCC: Camphor-*Crataegus* berry combination
CE: *Crataegus* fruit extracts
CHF: Congestive heart failure
CHM: Chinese herbal medicine
CM: *Crataegus microphylla*
DBP: Diastolic blood pressure
DP: Double product
EDHF: Endothelium derived hyperpolarizing factor
EGF: Epidermal growth factor
ERK: Extracellular signal-regulated kinase
FGF: Fibroblast growth factor
FMD: Flow mediated dilation
FMLP: Formyl-Met-Leu-Phe
HCAEC: Human coronary artery endothelial cells
HFC: Hawthorn fruit compound
HR: Heart rate
HUVECs: Human umbilical vein endothelial cells
I/R: Ischemia and reperfusion
IP3: Inositol 1,4,5-trisphosphate
LDL: Low-density lipoprotein
LDL-C: Low-density lipoprotein cholesterol
LVEF: Left ventricular ejection fraction
MAP: Mean arterial pressure
MWL: Maximal workload
NE: Neutrophil elastase
NO: Nitric oxide
NYHA: New York Heart Association
OA: Oleanolic acid
OPC: Oligomeric procyanidins
PHRPI: Pressure-heart rate product increase
PLA2: Phospholipase A2
QOL: Quality of life
RBCs: Red blood cells
SBP: Systolic blood pressure
SERCA: Sarcoplasmic/endoplasmic reticulum Ca^2+^ ATPase
SOCE: Store-operated calcium entry
TC: Serum cholesterol
TCR: Tincture of *Crataegus *
TG: Triglyceride
TXB2: Thromboxane B2
UA: Ursolic acid
Vcfc: Velocity of circumferential shortening
VSMCs: Vascular smooth muscle cell
VT: Ventricular tachycardia.
